# Hamam. in Hæmorrhoids

**Published:** 1883-01

**Authors:** H. D. Baldwin

**Affiliations:** Syracuse, N. Y.


### HAMAM. IN HÆMORRHOIDS.

H. D. Baldwin, M. D., Syracuse, N. Y.

Mrs. S., age about 60, came to me three years ago, while I resided in Montrose, or rather her son came to me for her, and described the following condition: His mother had had hæmorrhoids for forty years, and so bad as to prevent her being on her feet any length of time, because of the protrusion of tumors and severe hemorrhage, the blood often running down the limbs to the floor. Prescribed *Ham.* 1st dec. dilution, to be taken four pills one hour before each meal and on retiring, but told him that I thought that an operation would be required. The remedy was continued, with an occasional dose of *Sulphur*$^{30}$ for one year, as there was a gradual improvement. A few weeks ago, Nov. 8th, he came to me to say that although she had been working all summer, and been almost constantly on her feet, she had no return of hæmorrhoids or hemorrhage and considered herself well. The result of this was very gratifying to me, because, 1st, I had never met with a case of that length standing which yielded to internal remedies alone, and 2d, why do not all cases, when all symptoms are seemingly as well marked as they were in this, respond as favorably to medication? Most all cases of hæmorrhoids of any length of standing have to be removed by an operation, but I am thoroughly convinced that acute cases can be entirely removed by internal medication if the remedy is carefully selected.

